# Characterizing porous microaggregates and soil organic matter sequestered in allophanic paleosols on Holocene tephras using synchrotron-based X-ray microscopy and spectroscopy

**DOI:** 10.1038/s41598-021-00109-9

**Published:** 2021-10-29

**Authors:** Doreen Yu-Tuan Huang, David J. Lowe, G. Jock Churchman, Louis A. Schipper, Alan Cooper, Tsan-Yao Chen, Nicolas J. Rawlence

**Affiliations:** 1 School of Science/Te Aka Mātuatua, University of Waikato, Private Bag 3105, Hamilton, 3240 New Zealand; 2 Department of Ecology and Environmental Science, Umeå University, 901 87 Umeå, Sweden; 3 School of Agriculture, Food and Wine, University of Adelaide, Private Mail Bag No. 1 Glen Osmond, Adelaide, SA 5064 Australia; 4 South Australian Museum, Adelaide, SA 5000 Australia; 5 BlueSky Genetics, P.O. Box 287, Adelaide, SA 5137 Australia; 6 Department of Engineering and System Science, National Tsing Hua University, Hsinchu, 30013 Taiwan; 7 Otago Palaeogenetics Laboratory, Department of Zoology, University of Otago, P.O. Box 56, Dunedin, 9054 New Zealand

**Keywords:** Biogeochemistry, Environmental sciences

## Abstract

Allophanic tephra-derived soils can sequester sizable quantities of soil organic matter (SOM). However, no studies have visualized the fine internal porous structure of allophanic soil microaggregates, nor studied the carbon structure preserved in such soils or paleosols. We used synchrotron radiation-based transmission X-ray microscopy (TXM) to perform 3D-tomography of the internal porous structure of dominantly allophanic soil microaggregates, and carbon near-edge X-ray absorption fine-structure (C NEXAFS) spectroscopy to characterize SOM in ≤ 12,000-year-old tephra-derived allophane-rich (with minor ferrihydrite) paleosols. The TXM tomography showed a vast network of internal, tortuous nano-pores within an allophanic microaggregate comprising nanoaggregates. SOM in the allophanic paleosols at four sites was dominated by carboxylic/carbonyl functional groups with subordinate quinonic, aromatic, and aliphatic groups. All samples exhibited similar compositions despite differences between the sites. That the SOM does not comprise specific types of functional groups through time implies that the functional groups are relict. The SOM originated at the land/soil surface: ongoing tephra deposition (intermittently or abruptly) then caused the land-surface to rise so that the once-surface horizons were buried more deeply and hence became increasingly isolated from inputs by the surficial/modern organic cycle. The presence of quinonic carbon, from biological processes but vulnerable to oxygen and light, indicates the exceptional protection of SOM and bio-signals in allophanic paleosols, attributable both to the porous allophane (with ferrihydrite) aggregates that occlude the relict SOM from degradation, and to rapid burial by successive tephra-fallout, as well as strong Al-organic chemical bonding. TXM and C NEXAFS spectroscopy help to unravel the fine structure of soils and SOM and are of great potential for soil science studies.

## Introduction

The secondary clay minerals in soils are the reactive inorganic components that form aggregates in strong association with soil organic carbon (SOC)^1,2^, and the retention of soil organic matter (SOM) in small pores within soil aggregates has been proven to slow carbon turnover and its rate of decomposition^3–5^. Allophane is an Al-rich nanocrystalline aluminosilicate, formula (1–2)SiO_2_·Al_2_O_3_·(2–3)·H_2_O, that comprises hollow spherules ~ 3.5 to 5 nm in diameter. It has a very high specific surface area (up to ~ 1200 m^2^ g^−1^)^6–8^, enabling allophanic soils derived from tephra (volcanic ash), including Andisols, to adsorb much SOM (including via strong Al-SOM bonding) and to help stablize SOC^9,10^. The similar but Fe-rich nanocrystalline ferrihydrite, formula Fe_5_HO_8_∙4H_2_O, is also common in Andisols but typically in considerably smaller quantities than allophane unless on basaltic parent tephras^2,9^. The aggregation of allophane spherules generates fractal porous networks to retain and sequester considerable amounts of SOM which is spatially protected against degradation^11–15^. Although ferrihydrite likely stabilizes SOM in a similar way to allophane^1,2,9^, allophane is the main focus of our study.

A distinctive feature of many tephra-derived allophanic soils on stable sites is their multi-layered nature giving rise to pedostratigaphy^9,16^. Such soils are formed by upbuilding pedogenesis during which soil evolution occurs via topdown processes whilst tephras (including cryptotephras) are concomitantly added to the land/soil surface^16–18^. The thickness and frequency of tephra accumulation (and other factors) determine if developmental or retardant upbuilding, or both, takes place (nomenclature follows Johnson and Watson-Stegner^19^; Johnson et al.^20^; Almond and Tonkin^21^). One of the advantages of tephra layers is that, once identified, they provide isochrons to connect and synchronize sequences and to assign relative or numerical ages using tephrochronology^22^. Therefore the ages (or age ranges) of buried soils (paleosols) on multiple tephras are able to be dated.

The central North Island of New Zealand has huge stores of buried, allophane-rich paleosols developed on sequences of well-dated tephra beds^16,17,23–25^. The early Holocene of this region was dominated by extensive podocarp-broadleaf forest and warm and wet conditions followed generally by gradual drying and cooling^26–29^ with modest climatic fluctuations^30^. Thus, the slow carbon turnover rate in allophane-rich soils, and the abundance of buried allophanic paleosols on dated tephras in North Island, provide the opportunity to characterize the nature of stabilized SOM preserved in allophanic soils/paleosols in response to changes of environmental and climatic conditions.

Conventional studies of the nature of SOM have relied mainly on chemical extractions^31^. However, the extractable components could only partially represent the nature of SOM^32,33^. Modern spectroscopic techniques including ^13^C nuclear magnetic resonance (NMR), Fourier transform infrared (FTIR), pyrolysis gas chromatography-mass spectrometry (pyrolysis-GC/MS), and carbon near-edge X-ray absorption fine structure (C NEXAFS) spectroscopy, are used for characterizing SOM^34–37^ in order to more closely analyze in situ organic matter by avoiding artefacts derived from the extraction techniques.

**Figure 1:**
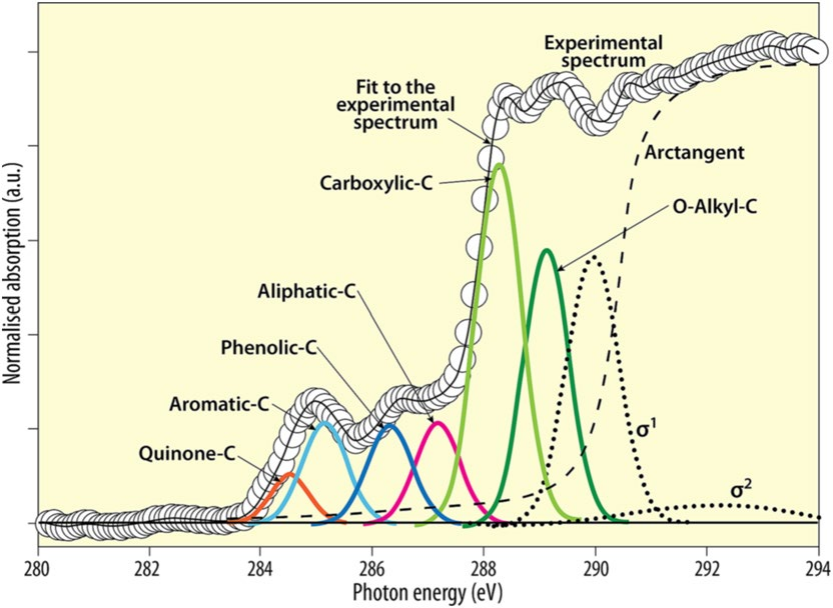
C *K*-edge NEXAFS spectra for humic substances extracted from clay fractions of a soil from Wushwush, Ethiopia, and the spectra deconvolution showing the transitions (multiple peaks) of various carbon functional groups (redrawn from Solomon et al.^34^, p. 110, with permission from the Alliance of Crop, Soil, and Environmental Science Societies, publishers of *Soil Science Society of America Journal*).

Paramagnetic metals in soils interfere with ^13^C NMR spectroscopy for SOM and thus hydrofluoric acid (HF) has been used to dissolve the majority of soil minerals before such analysis^38,39^. However, HF treatment results in the loss of water-soluble SOM that is held as organo-mineral complexes^39^. The investigation of the structural composition of SOM using FTIR spectroscopy is difficult also because of the overlap of absorption bands of organic matter and inorganic soil components^40^. Instead, C NEXAFS spectroscopy involves promotion of core electrons (in the K shell) to higher orbitals and allows monitoring of the emitted electrons and photons. Other soil components (e.g. clays and water) do not interfere with the analysis of carbon by C NEXAFS spectroscopy^5,34,40^. Figure 1 illustrates how different functional groups (multiple peaks) of soil humic substances contribute a characteristic spectrum. Kruse et al.^36^ also nicely documented the assignment of C K-edge XANES (NEXAFS) peak energy positions to C moieties.

Our objectives were to analyze the porous structure of allophanic microaggregates and functional groups of SOM preserved from past environments associated with allophanic (and ferrihydritic) tephra-derived Holocene paleosols to further understand carbon sequestration in Andisols and associated paleosols. We also wanted to evaluate the potential of C NEXAFS spectroscopy for soil evolution studies and paleopedology. From four sites in central North Island, we sampled a series of buried paleosols formed by developmental and/or retardant upbuilding pedogenesis on Holocene tephra layers, all formed under podocarp-broadleaf forest until only ca 700 calendar (cal.) yr ago (when Polynesian arrival led to partial deforestation), their ages established via tephrochronology^41^. Each paleosol represents an age range from ca 1100 to ca 12,000 cal. yr of pedogenesis (soil formation) at the land surface before burial. Just the organo-clay complexes were analyzed because only the clay fraction has a strong association with SOM and SOC retention in soils^1,2^, and the preserved SOM is held largely within microaggregates^42^. We first used high-resolution transmission X-ray microscopy (TXM) to examine the internal structure of the microaggregates to help envisage and explain the preservation of SOM within them. The aggregates were then analyzed by C NEXAFS spectroscopy to resolve the carbonaceous functional groups (also known as carbon speciation) of SOM of various ages, which is the first analysis to be undertaken on sequences of buried allophanic paleosols formed on Holocene tephras of known age.

## Materials and methods

### Stratigraphy and sampling of paleosols

**Figure 2:**
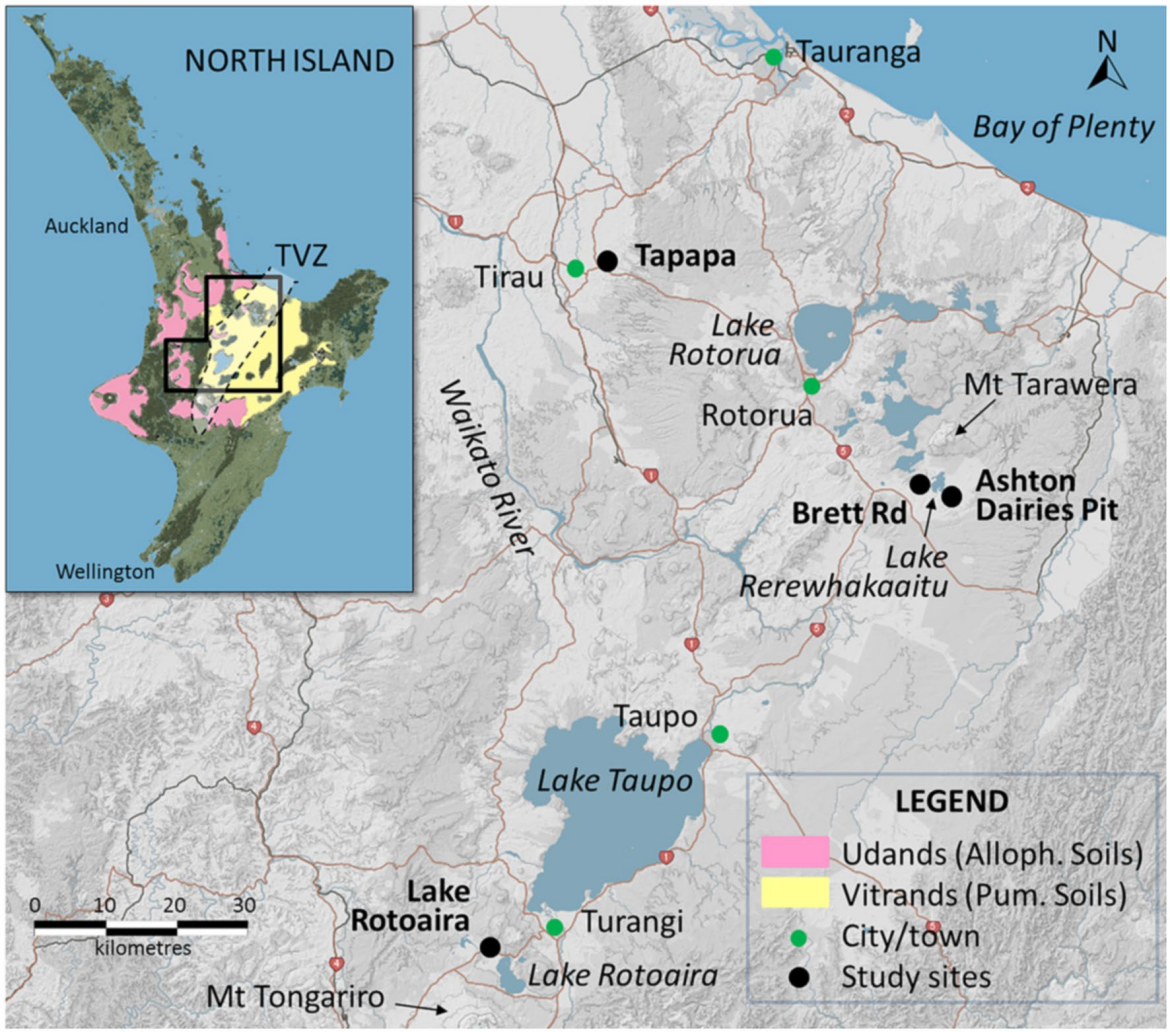
Map of central North Island, New Zealand, showing the locations of the four study sites in the Taupo-Rotorua region. The inset map shows the distribution of Andisols, mainly Udands (equivalent to Allophanic Soils in Hewitt^44^) and Vitrands (equivalent to Pumice Soils in Hewitt^44^), and the location of Taupo Volcanic Zone (TVZ) encompassing the volcanoes or volcanic centres that erupted the parent tephras of the soils and paleosols under study^16,17,24^. The maps were derived from the S-map Online (http://smap.landcareresearch.co.nz/home) and are licensed under CC BY-NC-ND 3.0 NZ, and the maps were modified for the indications of TVZ and study sites. State highways are numbered (red shields).

We selected allophane-rich soil material and paleosols developed almost all on Holocene tephra-fall deposits of both rhyolitic (i.e. with high silica content, ≥ ∼ 70 wt% SiO_2_) and andesitic (with intermediate silica content, ∼ 50–70 wt% SiO_2_) composition at four geomorphically-stable sites in central North Island (Fig. 2), two relatively close to the main volcanic sources (= proximal sites) and two farther away (= distal sites). At two sites—Brett Rd, Ashton Dairies Pit—the (now buried) soil on Taupo eruptives is formed on loose (i.e. non-welded), pumiceous Taupo ignimbrite, derived from a pyroclastic flow, rather than fall deposits^43^.

**Figure 3:**
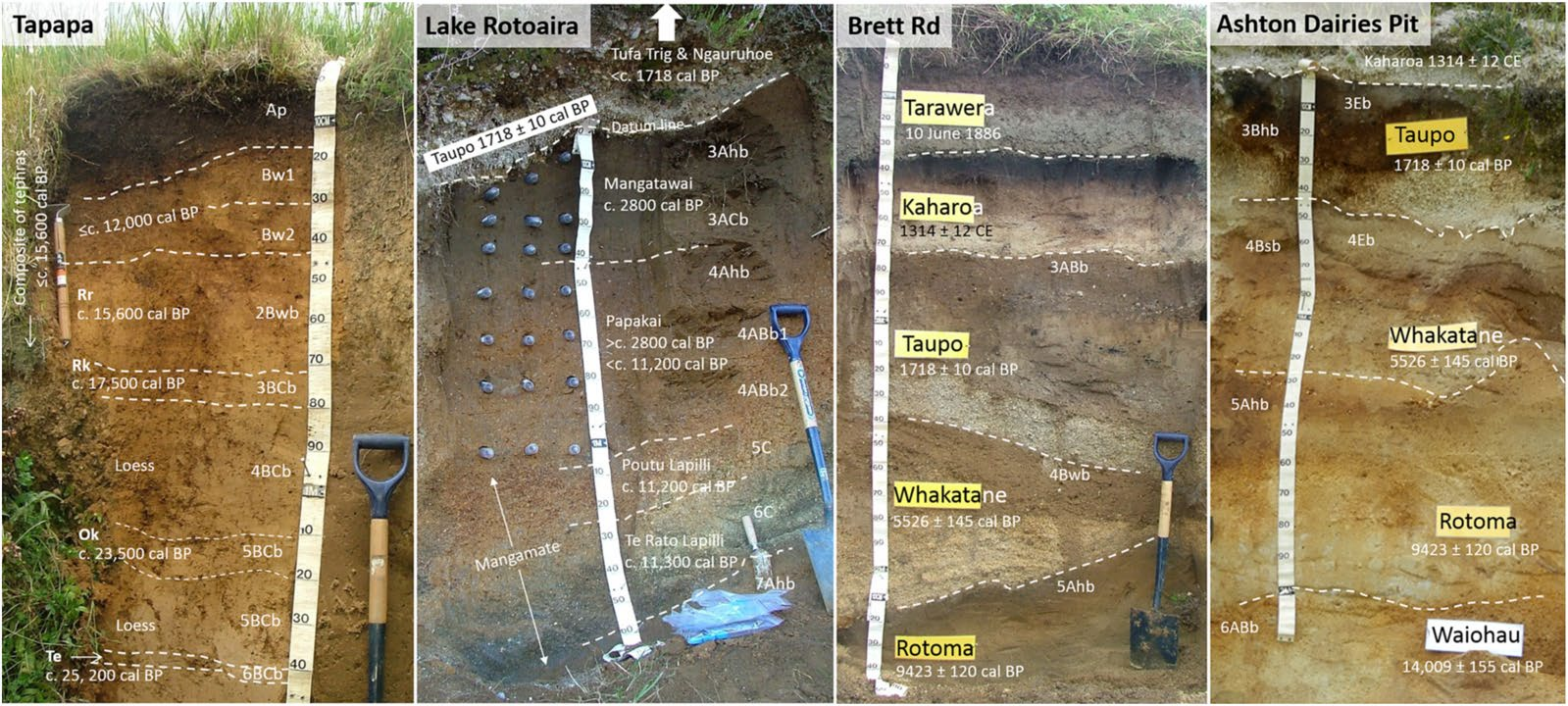
Photos of soil profiles showing soil horizons and their notation, and the stratigraphy and names and ages of parent tephras (ages were derived using tephrochronology) at Tapapa, Lake Rotoaira, Brett Rd, and Ashton Dairies Pit (Fig. 2). The suffix ‘b’ is used to denote an identifiable soil horizon with pedogenic features developed before its burial. Note that some of the buried soil horizons at Ashton Dairies Pit (3Eb, 3Bhb; 4Eb, 4Bsb) display morphologies associated with podzolization^2^. More details are given in Table 1 and Supplementary Sect. 1. *Rr* Rotorua tephra, *Rk* Rerewhakaaitu tephra, *Ok* Okareka tephra, *Te* Te Rere tephra, *BP* before present. For almost all of the Holocene, the soils carried a similar forest cover, namely podocarp-broadleaf forest (currently extant at the Lake Rotoaira site), which was temporarily interrupted by the deposition of thick tephra at Brett Rd and Ashton Dairies from time to time. Scale divisions on tape = 10 cm. Photos: D. J. Lowe.

The multi-layered soils at the two distal sites were formed mainly by developmental upbuilding: Tapapa (formed on a composite of mainly rhyolitic tephras), near Tirau; and Lake Rotoaira (formed on a composite of mainly andesitic tephras), near Turangi and close to Mount Tongariro (Fig. 2). The multi-layered soils at the two proximal sites were formed on rhyolitic tephras via retardant upbuilding: Brett Road and Ashton Dairies Pit, both near Mount Tarawera to the southeast of Rotorua (Fig. 2). Roadside tephra-soil profiles (sections) at Tapapa and Lake Rotoaira were incised ~ 50 cm laterally to remove any modern roadside plant material before sampling; the roadside profile at Brett Rd was incised ~ 1 m laterally; and the profile at Ashton Dairies was in a pit newly excavated using a mechanical digger. The stratigraphy and soil horizonation for each soil profile were established using tephrostratigraphy—see Supplementary Sect. 1—and conventional soil morphological examination (Fig. 3; Soil Survey Staff^45^ and Soil Survey Staff^46^). The approximate lengths of time the tephra materials at each site were at, or near, the land surface undergoing weathering and pedogenesis (by topdown soil processes forming soil horizons) before their burial by subsequent tephra(s) are reported in Table 1. These ages, or age ranges, for the paleosols were derived using tephrochronology.

**Table 1:** Depths, ages, and pH values of the soils and buried-soil horizons at the four sites, and their clay, allophane, ferrihydrite, and total organic carbon (TOC) contents. Notes for different symbols are: ^a^from equivalent buried soil horizons on the same tephras at nearby sites identified using pedostratigraphy and tephrochronology (from Lowe and Tonkin^18^, after Green^47^); ^b^below the datum line of the base of (non-welded) Taupo ignimbrite (Fig. 3); ^c^below the datum line of the base of Kaharoa tephra (Fig. 3); ^d^estimated likely value/range of values associated with similar horizons developed on equivalent Holocene andesitic tephras in the region^48,49^; ^e^estimated from equivalent buried soil horizons (identified using pedostratigraphy and tephrochronology) on the same tephras at nearby sites^48,49^. NA indicates data not obtained.

Site (horizon notation)	Depth	Age (range) of paleosol	pH (H_2_O)	Clay content (< 2 µm)	Allophane content (fine-earth basis)	Ferrihydrite content (fine-earth basis)	TOC (clay basis)
	cm	cal. yr BP		wt %	wt %	wt%	wt %
**Tapapa**							
Bw1	20–30	≤ ca 12,000	5.8	10.6	8.3	0.7	10.6
Bw2	30–40	≤ ca 12,000	5.9	6.8	10.4	0.8	7.7^a^
**Lake Rotoaira**							
3Ahb	0–20^b^	2800–1718	6.3	9.3	11.3	~ 2^d^	7.0
3ACb	20–40^b^	2800–1718	6.2	8.1	18.3	1.2	6.9
4Ahb	40–60^b^	11,200–2800	6.2	16.6	32.7	~ 2–3^d^	6.7
4ABb1	60–80^b^	11,200–2800	6.2	19.0	18.6	~ 2–3^d^	6.5
4ABb2	80–100^b^	11,200–2800	6.1	17.2	14.5	~ 2–3^d^	7.0
**Brett Rd**							
3ABb	70–80	1718–636	6.6	3.4	3.0	0.5^e^	11.9
4Bwb	140–150	5526–1718	6.8	3.9	5.3	0.5^e^	10.2
5Ahb	210–220	9423–5526	6.6	5.0	2.5	0.3	6.3
**Ashton Dairies Pit**							
3Bhb	20–30^c^	1718–636	6.5	4.7	2.1	0.5^e^	NA
4Bsb	60–80^c^	5526–1718	6.5	2.9	1.2	0.3	10.5
5Ahb	130–140^c^	9423–5526	5.5	1.1	4.8	0.8^e^	13.3^a^

Soil horizons at all sites were tested using the NaF-based allophane test in the field^44^ to confirm that they were allophanic (samples were later analyzed in the lab for allophane content). In total, 13 samples of buried allophanic soil horizons (paleosols) were collected (Table 1). They were sieved to obtain < 2 mm-size (fine-earth) fractions and stored at 4 °C in the dark for up to one month prior to clay extraction and analyses.

### Soil properties and extraction of organo-clay fractions

Soil pH values (solid/solution = 1/2.5) were measured in water following the method of Blakemore et al.^50^ (Table 1). Allophane contents of soil samples were estimated by oxalate-extractable Fe, Al, and Si as well as pyrophosphate-extractable Al^50,51^; ferrihydrite contents were estimated from oxalate-extractable Fe multiplied by 1.7^52^. Before extraction of clay fractions, visible root remnants were removed. To obtain clay fractions (particles < 2 µm), each sample was dispersed mechanically by prolonged shaking for 16 h with deionized (DI) water and 2-mm glass beads^53^, followed by sedimentation of particles > 2 µm according to Stokes’ Law and then suspended clay was extracted via a pipette. Andisols possess strong physical stability, thus size fractionation by shaking may result in incomplete disruption/dispersion of soil aggregate as compared with the sonification method^54,55^. Therefore, some stable aggregates > 2 µm formed with clay particles might not have been disrupted during shaking and were precipitated during sedimentation, thus we may have underestimated the quantity of clay in the soils we studied. However, the purpose of clay fractionation here was to acquire clay-size particles/aggregates for characterizing the clays and preserved SOM in them, and so any underestimation of clay fractions would be of little consequence.

The clay fractions were frozen quickly with liquid nitrogen and freeze-dried to preserve the nature of organic matter associated with the clay. The freeze-dried (organo-)clays were ground and analyzed in duplicate for total organic carbon (TOC). Although we did not test in this study, the exposure of samples to oxygen and light during all the treatments may have caused minor organic matter degradation thus some vulnerable functional groups in soils might be affected. Analysis of TOC was undertaken using a Leco TruSpec carbon/nitrogen analyzer. The pH values, clay, allophane, and ferrihydrite contents of fine-earth fractions collected from the paleosols are shown in Table 1, together with the TOC contents (of clay fractions) and stratigraphic and age information.

### Characterizing internal porous structure of allophane aggregates using TXM

To help envisage and explain the preservation of the SOM identified in the allophanic buried soils/paleosols, we used a synchrotron-based transmission X-ray microscope (TXM) which allows two dimensional (2D) imaging and 3D tomography at tuneable energies from 6 to 11 keV. The experiment was carried out at BL01B1 at the National Synchrotron Radiation Research Center (NSRRC) in Hsinchu, Taiwan. At the sample position, the expected photon flux is about 7 × 10^11^ photon/s/200 mA and the focused beam size is about 1 mm × 0.4 mm, and the current beamline allows images and 3D tomography acquisitions with 60-nm spatial resolution^56^. Many natural allophane microaggregates extracted from the Bw1 horizon of the soil at Tapapa (Fig. 3) were firmly adhered to silicon-free tapes to avoid movement of microaggregates during rotation. All the microaggregates were examined at 1.84 keV for silicon *K*-edge. 2D micrographs of each microaggregate were generated using TXM with 60 s exposure time to obtain micrographs of high resolution; the 3D tomography datasets were reconstructed based on sequential image frames taken with azimuth angle rotating from − 75° to + 75° to obtain 151 2D micrographs of the microaggregate, with shadow represents the silicate-based clay and the white areas were silicate-free inorganic compounds and pores.

The final 3D virtual structure (video view) of the microaggregate was generated from the tomographic dataset using the software Amira 5.01, Visage Imaging. The image alignment is the important procedure and critical to allow 3D tomography and 3D computed reconstruction. Only images from one microaggregate could be well aligned and thus we present only the 3D tomography and reconstruction of that microaggregate.

### Characterizing SOM sequestered by clays using C NEXAFS spectroscopy

C NEXAFS spectra for (organo-) clays and for pure indium foils (as sample carriers) were collected at beamline 24A1 at NSRRC. Typically, to normalize the signals from samples, the total electron yield from each sample is divided by the yield of a clean surface (namely I_0_) measured concurrently with the sample of interest, but often the C accumulation in the beamlines leads to false I_0_ signal for C analysis. Beamline 24A1 at NSRRC has a specific system that allows I_0_ to be obtained by X-ray travelling through a golden grid which had been set up in the beam path in the ionization chamber ahead of the analytical chamber. The golden grid is coated in-situ by ionized gold atoms ejected from a gold stick during a sputtering process before all the experiments are carried out. The signal derived when the beam passes through the just-coated golden mesh is designated to be carbon-free, and the interference of carbon accumulating in the beam path with spectra normalization is therefore eliminated. The beamline 24A1 was used to obtain high-resolution C 1 s X-ray photoelectron spectroscopy (XPS) spectra and C NEXAFS spectra^57,58^, indicating that the beamline is free from significant carbon accumulation/contamination and is thus suitable for C X-ray absorption spectroscopy (XAS).

Fluxes of photons 5 $\times$ 10^11^ to 1 $\times$ 10^10^ per second were admitted to the chamber, and the beamline produced soft X-rays (energy range < 5 keV) so that the energy was tuneable over 10–1500 eV and allowed a focussed beam size of 0.7 by 0.3 mm. The end station comprised three chambers, including a pre-chamber, a transfer chamber, and a main analytical chamber. The last is under very high vacuum (< 1 ×10^–8^ torr). For C NEXAFS spectroscopy with high resolution, the beamline grating was set to 400 mm^−1^, and the positions of two beamline silts were set to − 200 and − 20 on the dial to minimize the light source and focus the beam.

For sample preparation, disposable gloves were worn and tools were cleaned with acetone to avoid carbon contamination from personnel or preparation. An extracted organo-clay fraction from each soil sample was pressed into indium foil (0.5 mm thick, manufactured by Puratronic^®^, 99.9975%) to be conductive so that the excited electrons could be transmitted from the sample surface to the detector. The surface of indium foil was fully covered with sample to avoid any potential extraneous carbon contribution from the indium foil itself, and the indium foil with attached sample was flattened with clean glass rods. The flattened indium foil containing the sample was attached to a clean sample holder, and the holder was placed into the pre-chamber for degassing for 2 h and then transferred to the main analytical chamber.

The C NEXAFS spectroscopy was performed with the X-ray energy set to increase from 275 to 340 eV with a step of 0.035 eV (2000 dwell points in total, 1 ms dwell time per point), and it took approximately 3 min to obtain one spectrum. Three spectra were collected for each organo-clay sample based on total electron yield and partial electron detection modes with an electron energy analyzer (SPECS PHOBOIS 150). The photon energy was calibrated by setting the position of first valley of I_0_ collected from every scan to be 284.2 eV that corresponded with the first peak of graphite at 285.5 eV. The replicate C NEXAFS spectra for each sample were merged, processed, baseline-corrected, and normalized using the Athena program, an interface to IFEFFIT (version 1.2.11). The full spectra including post-edge region up to 340 eV were used for normalization (see an example in Supplementary Fig. SM1A). In our study we only present the spectra of total electron yield as they provided better resolution with high electron yield in our case, and we show spectra of samples between 280 and 310 eV because the peaks in this region representing various C functional groups were identified according to the X-ray energies.

## Results

### Porous internal structure of an allophane microaggregate from soil at Tapapa

**Figure 4:**
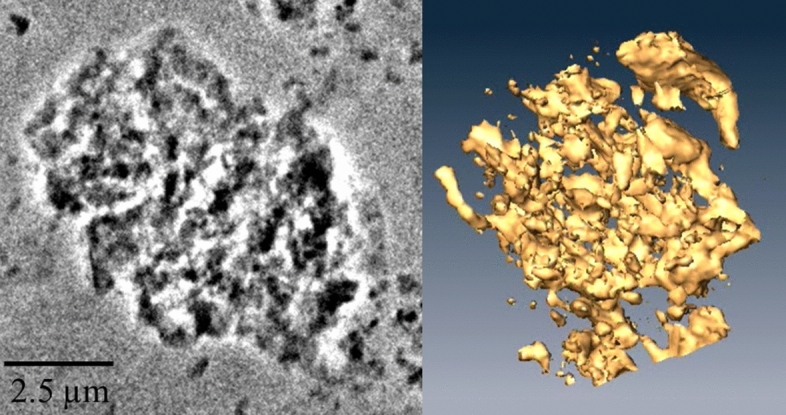
(Left) A 2D micrograph of a natural allophane-dominated microaggregate (extracted from the Bw1 horizon of the soil at Tapapa) obtained using TXM. Dark shadow represents the absorption of 1.84 keV (silicon *K*-edge) and thus the structure/shape of silicate-based clay, and the white areas are silicate-free compounds (potentially ferrihydrite) and pores. (Right) A 3D virtual reconstruction of the microaggregate obtained from 3D tomography datasets, showing its high porosity and high degree of pore network tortuosity (see Huang et al.^41^). The scale is the same for the two images, but the images (stills) show the microaggregate from different azimuth angles. Video views of the 2D image and the coloured 3D reconstruction are available as supporting materials. The 3D virtual structure of the microaggregate was generated from the tomographic dataset using the software Amira 5.01, Visage Imaging (https://visageimaging.com).

The 2D and 3D images of the examined microaggregate are presented in Fig. 4. The dark shadow in Fig. 4 (left) represents the absorption of silicon *K*-edge and thus the structure of silicate-based clay, and the white areas are the locations of silicate-free compounds (such as ferrihydrite and organo-metal complexes) and pores. The 3D virtual reconstruction of the allophane microaggregate showed that it was highly porous and comprised many sub-microaggregates or nanoaggregates (defined as ≤ 100 nm in diameter: Huang et al.^42^).

### Carbon from indium foil and authentic carbon signal from Beamline 24A1

**Figure 5:**
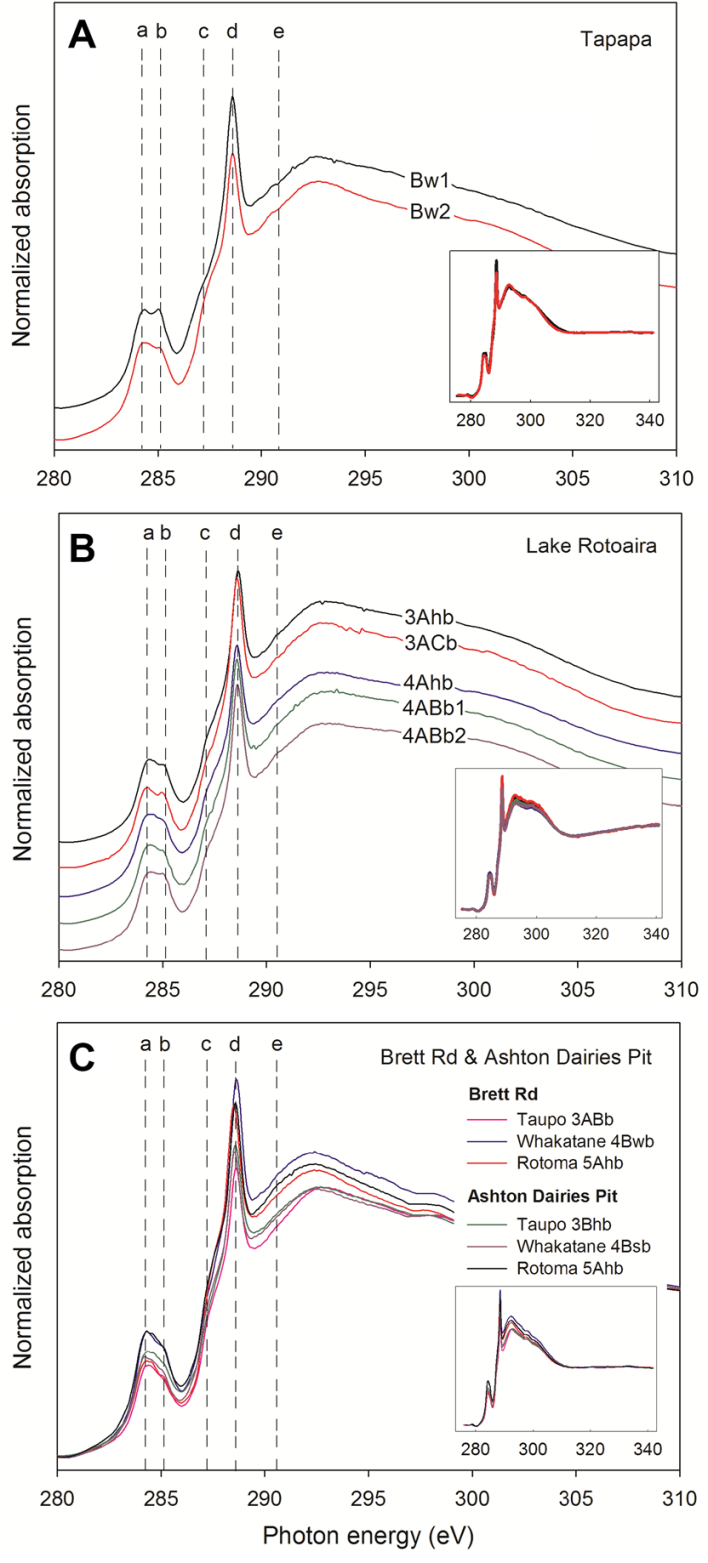
C NEXAFS spectra for organo-clays in allophanic paleosols. (**A**) Clays extracted from the Bw1 horizon (20–30 cm depth) and from the Bw2 horizon (30–40 cm depth) above Rotorua (Rr) tephra at Tapapa. (**B**) Clays extracted from the five soil subhorizons denoted 3Ahb and 3ACb (representing ca 1082 years of soil formation), and 4Ahb, 4ABb1, and 4ABb2 (representing up to ca 8400 years of soil formation) at Lake Rotoaira. (**C**) Clays extracted from upper allophanic soil horizons on Taupo tephra (representing ca 1082 years of soil formation), Whakatane tephra (representing ca 3808 years of soil formation), and Rotoma tephra (representing ca 3897 years of soil formation) at Brett Rd and Ashton Dairies Pit. Spectral features identified by the vertical lines correspond to carbon in (a) quinonic, (b) aromatic, (c) aliphatic, (d) carboxylic/carbonyl, and (e) carbonyl/carbonate functional groups (Fig. 1). Note there were cartographic shifts of spectra in both (**A**) and (**B**) graphs to show each spectrum clearly, but no shift in graph (**C**) in order to observe the difference of intensity of X-ray absorbance between samples collected from the two near-adjacent sites. The small insets show the full spectra including pre-edge and post-edge regions to indicate proper spectrum normalization.

The C NEXAFS spectrum for indium foil (99.9975% purity, used as sample carrier) showed that carbon from the indium foil was characterized by quinonic and carboxylic/carbonyl functional groups (Supplementary Fig. SM1A), which, despite the safeguards noted earlier (“Characterizing SOM sequestered by clays using C NEXAFS spectroscopy”), could be from the impurities when the indium foil was refined and manufactured or from extraneous carbon adsorbed on the indium foil before use. We further examined other carbonaceous materials, such as biochars and non-allophanic soil materials, and found different carbon functional groups (Supplementary Fig. SM1B,C). Quinonic carbon in particular was not observed in biochar samples, and there was significant loss of carboxylic/carbonyl groups in three samples. Thus, we concluded that the carbon signals from indium foil did not affect the analysis of soil mounted on the surface of indium, and that indium foil was a suitable holder for soil samples. Moreover, the spectra from our allophanic paleosols and from indium foil exhibited different baselines and intramolecular resonances (over the 290–296 eV region) (see Supplementary Fig. SM1A and Fig. 5), and so the carbon signal from samples could not have originated from the indium foil.

### Nature of SOM in allophanic clay fractions of Holocene tephra-derived soils/paleosols at the four sites

The clay fractions from allophanic buried soils/paleosols contain 6.5–13.3% TOC. At the developmental upbuilding sites in Tapapa and Rotoaira, we found very similar C functional groups compositionally and proportionally (Fig. 5A,B). The C NEXAFS spectra were mainly characterized by quinonic (284 eV), aromatic (285 eV), and carboxylic/carbonyl (288.5 eV) carbon, although the clay, allophane, and TOC contents of the soils varied markedly (Table 1). The results show that the structures of preserved SOM in clay fractions of paleosols on Holocene tephras of different ages (Fig. 3, Table 1) have remained the same, and that clay and allophane contents, and time, have not significantly affected the constituent functional groups of the SOM adsorbed by clays in these paleosols.

The paleosols at the retardant upbuilding sites, Brett Road and Ashton Dairies Pit, are each developed on the same set of Holocene tephras, including Taupo, Rotoma, and Whakatane tephras. The clay-attached organics in paleosols on each of these tephras at the two sites, despite some podzolic (strongly acidic leaching) soil morphological differences noted earlier (Fig. 3), still have similar spectral characteristics (Fig. 5C), including substantial amounts of carboxylic/carbonyl groups (> 40%) at 288.6 eV and relatively small contributions of quinonic (284.3 eV), aromatic (285 eV), and aliphatic (287.1 eV) groups despite differences in paleosol ages and depths (Fig. 3). As at Tapapa and Lake Rotoaira, these similarities indicate that the structures of preserved SOM in the clay fractions of Holocene paleosols of different ages have remained the same despite the differences in clay and allophane (and ferrihydrite) content and age, and the podzolization evident at Ashton Dairies Pit (Supplementary Sect. 1.3).

## Discussion

### Adsorptive clays, porous aggregates in allophanic soils/paleosols and upbuilding pedogenesis allow preservation of SOM

Previously the high surface area and the hydroxyl groups of allophane and iron (hydr)oxide (ferrihydrite) in tephra-derived soils and Andisols were considered to be one of the keys for SOM/SOC sequestration^59–61^. Basile-Doelsch et al.^61^ demonstrated that 83% of the organic matter in a buried tephra-derived soil was associated with such minerals and also they suggested microaggregates of organomineral complexes were preserved. Studies additionally showed that the intermediate-density fraction (where the majority of allophane and ferryhydrite are located in soil) in an Andisol could not explain SOC sorption onto the surface solely and so the role of organo-mineral aggregation was additionally suggested^55,62^. A later study based on the surface chemistry and adsorption capacity of synthetic allophane spherules showed that only 20% of organic compounds (presented as DNA molecules) were adsorbed on the surface of allophane and almost 80% of the organic compunds were held “physically” within small pores of allophane microaggregates and nanoaggregates^42^. These studies thus suggest that the nanocrystalline clays govern SOM sequestration in Andisols by both surface sorption and aggregation/entrapment.

The considerable quantaties of allophane in most of the soils/paleosols we studied have contributed to SOM stabilization in the soils. Ferrihydrite, although relatively minor in quantity (Table 1), would also be expected to contribute to SOM sequestration (following Basile-Doelsch, et al.^61^) because it, like allophane, has a large reactive surface area (up to ~ 500 m^2^ g^−1^)^9^. Our 3D tomography of a microaggregate from the Bw1 horizon of the allophanic soil material at Tapapa further shows the aggregation of clays and the vast internal porous structure within the microaggregate, and such an extent of fractal nano-pores (described as a “nanolabyrinthic” pore distribution with a high degree of tortuosity) allows storage of SOM and decreases SOM bioavailability and carbon turnover rate^4,11,42^. The synchrotron-based TXM enables the analysis of soil microstructure, but the reconstruction of the structure at nano-scale could be limited because of imperfect image alignment. Thus, the development of better alignment approaches^56^ may enhance the use of TXM for soil science studies at the molecular/nano-scale range. Ongoing tephra accretion then causes the land surface to rise so that the once-surface horizon becomes more deeply buried and hence increasingly isolated from the modern (surficial) organic cycle. Consequently, the effects of pedogenesis (parent material transformation) become negligible or nil: in the case of incremental, thin tephra/cryptotephra deposition (i.e. developmental upbuilding), the isolation is gradual whereas in the case of sudden thick tephra deposition (i.e. retardant upbuilding) it is abrupt or paroxysmal (these concepts were first described by Taylor^63^ and Hopkins et al.^25^; see also Schaetzl and Sorenson^64^). Although downward leaching of mobile, younger SOM from the surface through the profiles occurs, we contend that such SOM would not be well protected chemically unless it was able to be encapsulated into the nanolabyrinthic pore network, which, however, would be largely occupied by older SOM encased physically during past periods of soil formation that included weathering and dissolution of abundant volcanic glass by hydrolysis (where the proton donor was usually carbonic acid, together with organic acids especially at Ashton Dairies Pit) and the synthesis (neoformation) of the dissolution products to form clays near the soil/land surface^65^.

Thus, much of the SOM adsorbed and preserved chemically and physically by allophane in clay fractions of the buried paleosols on Holocene tephras in New Zealand is, we suggest, derived mainly from past environments when the organo-allophane micro/nanoaggregates were formed at or close to the (paleo) land surface prior to their burial. This conclusion is supported by the earlier findings on ancient plant DNA extracted from a buried paleosol on Rotoma tephra at the Brett Rd site^66^.

### Similarity of carbon functional groups of SOM in paleosol clay fractions

In our study, the shapes of spectra for SOM in clay fractions showed no significant increase nor decrease in intensity of specific C functional groups between samples, revealing the high similarity of stabilized C in the clay fractions of the allophane-rich buried soil horizon/paleosols. Although Heymann et al.^32^ showed that the proportions of aromatic C and *O*-alkyl C in alkaline extracts from soils at different depths could be varied, the SOM in the non-treated clays from our paleosols shows the same carbon functional groups. The predominance of carboxylic/carbonyl carbon in the allophanic soils/paleosols examined in our study may result from (1) a strong association of carboxylic/carbonyl groups of humic substances with Al–OH defects on the surface of allophane by ligand exchange^13,59,67^, or (2) oxidation of organics and microbial activities in uppermost soil horizons (at the land surface under an active organic regime)^68^ in the past and before the land surface was buried by new tephra deposits, or both. The regional C NEXAFS spectra over a stable microaggregate selected from a surface horizon of an Andisol also suggested that the microbial-derived amide and carboxylic C were the main forms of C in organo-mineral (nano)complex^69^. We therefore infer that the SOM originated at or near the land surface during upbuilding pedogenesis.

As soil genesis and hydrolysis-dominated weathering began in a newly-deposited tephra uppermost in the soil profile, allophane (and subordinate ferrihydrite) formed quickly^7^ and SOM was sequestered on spherules and in nano-pores within microaggregates. As noted above, ongoing tephra deposition (incrementally and/or suddenly) caused the land surface to rise so that horizons formerly at the surface were gradually or suddenly buried and hence over time became increasingly isolated from inputs by the modern organic cycle and near-surface processes. The presence of quinonic carbon, especially sensitive to oxygen and light, is indicative of the exceptionally strong protection of SOM by clays in allophanic paleosols, and attributable both to a tortuous nano-pore network amidst allophane micro-and nanoaggregates that encapsulates and shields the relict SOM from degradation, and to rapid burial by successive tephra-fall deposition. The occlusion, together with rapid burial, would help cut out light (which is unlikely to penetrate beyond ca 10 mm: Tester and Morris^70^) and thus reduce photodegradation of quinonic carbon by solar irradiance^71^. The mean rates of tephra accretion at Brett Rd and Ashton Dairies Pit are ~ 25 mm per century, and those at Tapapa and Lake Rotoaira are ~ 5 to 12 mm per century, respectively (after Lowe^72^), and so, on average, surface horizon components typically would be buried beyond light penetration within just decades. The clay-associated SOM (as expressed by the carbon functional groups) in the paleosols at each of the four study sites thus likely derived from soil processes operating from early to late Holocene and has not been modified by modern surface processes, or by diagenesis, after burial.

An early study in southern Italy also showed no differences in the chemical composition and structure of the humic substances extracted from six paleosols formed on a sequence of pyroclastic deposits dating from ca. 30,000 to 7000 cal. yr BP, despite the difference in age^73^, and this study concluded that the paleosols appear to be “closed systems from the geochemical point of view”. Similarly, with the use of solid-state NMR spectroscopy, the SOM in 50, 100, 300, 700, and 2000 year-old soils collected from a paddy soil chronosequence in China showed similar compositions of carbon functional groups^74^. Kleber et al.^75^ also showed that old, preserved SOM adsorbed by clays does not comprise a particular type of carbon, but a 680-year-old Inceptisol (a weakly developed soil with a Bw horizon: Soil Survey Staff^46^) sequestered a large proportion of alkyl carbon (aliphatic functional groups) that can be metabolized easily.

### Presence of quinone as an indicator of bio-signal preservation in allophanic soils

In comparison with published C NEXAFS spectra for organic matter in other soils or soil aggregates^34,75,76^, our New Zealand allophanic buried soil horizon/paleosols studied here all contained a distinct amount of quinonic carbon (over 284 eV region). Natural quinones are common constituents of bacterial plasma membranes^77,78^ and of pigments of chloroplasts^79^, which are involved in cellular respiration and photosynthesis. Among natural quinones, isoprenoid quinones have been used as taxonomic markers^78^ and are one of the most important groups of quinones because of their functions. Isoprenoid quinones are composed of a hydrophilic head group and an apolar isoprenoid side chain, giving the molecules a lipid-soluble character^80^. These quinones are hydrophobic and particularly susceptible to breaking down in well-drained alkaline conditions and are photo-oxidized rapidly in the presence of oxygen and strong light^81,82^. Naturally, the quinonic ring (an unsaturated ring containing two –C=O groups) undergoes reversible reduction, leading to more stable quinol ring (an unsaturated ring containing two –OH groups)^80^. Therefore, quinonic carbon is labile and highly susceptible to degradation and transformation in soils, which explains the absence or trace presence only of quinonic carbon in most soils examined in previous studies^5,34,76^. Hence, the presence of quinonic carbon in some soils has been attributed to its occlusion and thus protection within organo-aggregates—for example, Solomon et al.^83^ mapped the carbon functional groups of an ultrathin section of a soil microaggregate and showed the quinonic carbon occurred only in the inner and intermediate regions of the aggregate.

The encapsulation of organic matter within labyrinthic pore networks amidst mainly allophane (± ferrihydrite) micro-and nanoaggregates, readily envisaged from the 3D microtomographic image in Fig. 4, allows such organic matter to be occluded and thus remain intact despite strongly acidic leaching and oxidizing conditions at our sites (cf. Chevallier et al.^11^; Huang et al.^66^). The good preservation of the bio-signal in allophane-rich soils is evidenced by the presence of quinones in the soils, and thus the allophane-rich paleosol archives are of great potential for future studies in reconstructing past environments via lipid biomarker or ancient DNA analyses.

## Conclusions

1. The synchrotron-based TXM allowed us to obtain the first visualization and reconstruction of the vast network of internal nano-pores, i.e. nanolabyrinthic pore structure, within a microaggregate extracted from tephra-derived allophanic soil material (together with minor ferrihydrite) of Holocene age. This highly porous, tortuous nano-scale network allows carbon to be tightly entrapped, and the ongoing, rapid burial and hence subsurface isolation of the soil horizons (via upbuilding pedogenesis), together help to explain the high SOM sequestration and slow carbon turnover rate in tephra-derived soils (see (3) below for a summary of the mechanism).
2. The SOM associated with mainly allophanic clay fractions in the buried soil horizon/paleosols, ranging in age from ca 12,000 to 1718 cal. yr BP, was dominated by carboxylic/carbonyl functional groups with subordinated amounts of quinonic, aromatic, and aliphatic groups. All samples from four study sites exhibited similar compositions despite differences in clay and allophane contents, stratigraphic position (depth of burial), age, parent-tephra composition (andesitic versus rhyolitic), rainfall (Lake Rotoaira > Tapapa, Brett Rd, Ashton Dairies), and dominant mode of soil genesis (developmental versus retardant upbuilding). The dominant carboxylic/carbonyl functional groups could be ascribable to close binding between these functional groups and allophane (± ferrihydrite) that is stronger than for other groups.
3. We envisage that the SOM (as expressed by the carbon functional groups) originated at or near the land surface via upbuilding pedogenesis and weathering dominated by hydrolysis in free-draining tephra layers. As soil genesis began in a newly-deposited tephra bed at the soil/land surface, allophane (± ferrihydrite) was precipitated from weathering-derived solutes^2^ and formed micro- and nanoaggregates, physically sequestering contemporary SOM from the modern (surficial) organic cycle. Ongoing tephra deposition then caused the land surface to rise so that previous-surface (A) horizons became increasingly divorced from the modern organic cycle through their ever-deepening burial, eventually forming isolated buried soil horizons or paleosols. The SOM in the paleosols at each of the four study sites thus derives from processes operating from early to late Holocene, and not from modern surface processes nor from diagenesis.
4. In comparison with published C NEXAFS spectra for SOM in other soil materials, our allophanic paleosols contained a distinct amount of quinonic carbon over 284 eV region, indicating strong preservation of bio-signals in these soils. Thus allophanic (± ferrihydrite) paleosol archives are of great potential for paleoecological studies via biomarker (i.e. lipid or DNA) analyses.
5. Our study suggests that the TXM possesses a great potential for study in soil structure at micro- and nano-scales, and that the C NEXAFS spectroscopy is an important method for evaluating the fine structure of SOM in order to study the persistence and change of SOM in response to environmental change.

## Supplementary Information

Supplementary Information.

Supplementary Video 1.

Supplementary Video 2.

## Data Availability

All the soil chemistry data as well as the TXM tomographic images and C NEXAFS spectra are available.

## Abbreviations

asl: Above sea level
AP: Ashton Dairies Pit
BP: Before present (‘present’ is 1950 in the radiocarbon [^14^C] timescale)
BR: Brett Rd
cal.: Calendar or calibrated
C NEXAFS: Carbon near-edge X-ray absorption fine structure
DNA: Deoxyribonucleic acid
DI: Deionized
FTIR: Fourier transform infrared
NMR: Nuclear magnetic resonance
NSRRC: National Synchrotron Radiation Research Center
Ok: Okareka tephra
pyrolysis-GC/MS: Pyrolysis gas chromatography-mass spectrometry
Rk: Rerewhakaaitu tephra
Rr: Rotorua tephra
SM: Supplementary materials
SOC: Soil organic carbon
SOM: Soil organic matter
Te: Te Rere tephra
TXM: Transmission X-ray microscopy
XAS: X-ray absorption spectroscopy
XPS: X-ray photoelectron spectroscopy
yr: Year(s)
