# Supplementary material for: Characterizing porous microaggregates and soil organic matter sequestered in allophanic paleosols on Holocene tephras using synchrotron-based X-ray microscopy and spectroscopy
Source: Sci Rep. 2021 Oct 29;11:21310. doi: 10.1038/s41598-021-00109-9 (PMC8556322; doi:10.1038/s41598-021-00109-9)
Supplement: Supplementary file 1 — Supplementary Information. [file 41598_2021_109_MOESM1_ESM.docx]

**Supplementary materials** for D. Y.-T. Huang, D. J. Lowe, G. J. Churchman, L. A. Schipper, A. Cooper, T-Y. Chen, & N. J. Rawlence, “Characterizing porous microaggregates and soil organic matter sequestered in allophanic paleosols on Holocene tephras using synchrotron-based X-ray microscopy and spectroscopy”

**1. Detailed information about study sites**

- 1. *Tapapa*

This soil, exposed on a privately-owned farm-track cutting just off Tapapa Road near Tirau (Fig. 2 of original article), has developed mainly by developmental upbuilding on a composite of distal thin rhyolitic tephra-fall beds and tephric loess deposits laid down incrementally (location: 37° 59’ 41.01” S, 175° 52’ 59.95” E, elevation 238 m above sea level [asl]). (Models of upbuilding pedogenesis are described by Hewitt et al.,^1^.) The soil stratigraphy shown in Fig. 3 and Table 1 is based on Pullar and Birrell^2^, Lowe^3,4^, and Lowe et al.^5,6^; most ages are from Lowe et al.^7^ with Peti et al.^8^ providing the age for Okareka tephra. The soil is a Medial, thermic Typic Hapludand (Soil Survey Staff^9^). Mean annual rainfall is ~1524 mm^10^. Two soil samples were collected from upper subsoils, namely the Bw1 (20−30 cm depth) and Bw2 (30−40 cm depth) subhorizons. The soil materials for these samples derive from a composite of weakly weathered tephras ≤ca 12,000 cal. yr BP in age that overlie Rotorua tephra (Fig. 3) and thus have relict properties (hence, strictly, they represent a ‘relict’ rather than a ‘buried’ paleosol using the nomenclature of Valentine and Dalrymple^11^; Bronger and Catt^12^. Current vegetation is pasture.

- 1. *Lake Rotoaira*

The soil occurs on State Highway 47 (Te Ponanga Saddle Road) near Lake Rotoaira (Fig. 2) (location: 39° 01’ 07.08” S, 175° 41’ 38.65” E, 580 m asl). The buried subsoil horizons of interest to us, depicted in Fig. 3, have formed mainly by developmental upbuilding pedogenesis on a composite of thin andesitic tephra-fall beds that were deposited incrementally since the fall of Mangamate tephra (the latter also known as Pahoka–Mangamate sequence: Nairn et al.^13^. The horizons we sampled are overlain by ca 0.8 m of non-welded rhyolitic Taupo ignimbrite^14^, the base of which provides the zero datum line for depth measurements, and (at the land surface) younger andesitic fallout tephras ca 0.3 m in thickness. These andesitic post-Taupo deposits thus indicate that both retardant as well as developmental upbuilding pedogenesis have occurred at this site. The soil stratigraphy and ages of tephras for the site follow Topping^15^, Donoghue et al.^16^, Moebis et al.^17^, and Hogg et al.^18,19^ (see also Nairn et al.^13^; Voloschina et al.^20^). The soil profile in its entirety − from the land surface − is classified as an Ashy/pumiceous, glassy, mesic Typic Udivitrand^9,21^, with the buried soil horizons (as defined by Soil Survey Staff^22^) beneath the Taupo ignimbrite we examined having a high allophane content (Table 1) and thus strong andic (allophanic) soil properties^23^. Mean annual rainfall is ~1944 mm^10^. We sampled the 3Ahb and 3ACb subhorizons (on Mangatawai tephra ca 1718 to ca 2800 cal. yr BP) and the 4Ahb, 4ABb1, and 4ABb2 subhorizons (on Papakai tephra ca 2800 to 11,200 cal. yr BP) in the lower part of the ca 4-m-high section using Taupo ignimbrite (ca 1718 cal. yr BP) as the upper datum and Poutu Lapilli (ca 11,200 cal. yr BP) as the lower one (Fig. 3). The periods the soil horizons developed at the land surface were ca 1082 cal. years (3Ahb, 3ACb) and up to ca 8400 cal. years (4Ahb, 4ABb1, 4ABb2) (Table 1). Current vegetation is native podocarp-broadleaf forest.

- 1. *Brett Rd and Ashton Dairies Pit*

The soil on *Brett Rd* near Mount Tarawera (location: 38° 17’ 53.53” S, 176° 28 52.83” E, elevation 452 m asl) (Fig. 2) has developed by retardant upbuilding on five relatively thick rhyolitic tephra beds (all fall beds except Taupo ignimbrite) as marked in Fig. 3. The soil stratigraphy is based on Cole^24^, Lowe et al.^5^, McDaniel et al.^23^, and Hartemink et al.^25^; ages are from Lowe et al.^7^. Mean annual rainfall is ~1464 mm^10^. We sampled the 3ABb subhorizon (on Taupo tephra ca 1718 cal. yr BP), the 4Bwb subhorizon (on Whakatane tephra ca 5500 cal. yr BP), and the 5Ahb subhorizon (on Rotoma tephra ca 9400 cal. yr BP).

The soil at the *Ashton Dairies Pit* (on a privately-owned farm off Ash Pit Road, location: 38° 18’ 09.82” S, 176° 32’ 32.01” E, 444 m asl), with a mean annual rainfall ~1428 mm^10^, is very similar stratigraphically and in origin (retardant upbuilding) to the nearby Brett Rd profile. At Ashton Dairies, however, the Taupo tephra (comprising a basal fall bed overlain by non-welded pumiceous ignimbrite) is overlain by a thicker deposit (ca 1.45 m) of Kaharoa tephra (its base provides the zero datum line for depth measurements in Fig. 3) and (at the surface) ca 0.45 m of Tarawera tephra. Further, several of the buried soil horizons on the Taupo and Whakatane tephras at Ashton Dairies Pit (Fig. 3) show distinct morphological features associated with podzolisation^6^, an acidic soil-leaching process manifested as pale, enleached E horizons (denoted albic horizons) and as very dark brown to dark reddish-brown, humus- and/or sesquioxide-enriched Bh or Bs horizons (denoted spodic horizons)^9,25^. For example, in Fig. 3, the profile at Ashton Dairies Pit shows a distinct grey albic horizon (denoted 3Eb) above the spodic 3Bhb horizon on Taupo tephra, and the uppermost horizon on the Whakatane tephra is also pale in colour and qualifies as an E horizon (denoted 4Eb) (see also Hartemink et al.^25^, p. 137). Consequently, uppermost B horizons on these tephras, rather than E horizons, were sampled: the 3Bhb subhorizon (on Taupo tephra ca 1718 cal. yr BP), the spodic 4Bsb subhorizon (on Whakatane tephra ca 5500 cal. yr BP), and the 5Ahb subhorizon (on Rotoma tephra ca 9400 cal. yr BP) (Table 1). At both sites, the amount of time the soil on Taupo tephra developed at the land surface was ca 1082 years (1718 minus 636 years [or AD 1314 minus 232]), Whakatane ca 3808 cal. years (5526 minus 1718 years), and Rotoma ca 3897 cal. years (9423 minus 5526 years) (Table 1). Both soils at Brett Rd and Ashton Dairies Pit are Fine-loamy/medial, mixed/glassy Typic Udivitrands^9^. Current vegetation at both sites is pasture.

- 1. *Ages of soil horizons (paleosols)*

The ages, or age ranges, for the paleosols at the four study sites were derived using tephrochronology (see Figure 3, Table 1). The ages/age ranges reported in Table 1 represent the amount of time the tephra materials at each site were at or near the land surface undergoing weathering and pedogenesis (forming soil horizons by topdown soil processes) before their burial by subsequent tephra deposition.


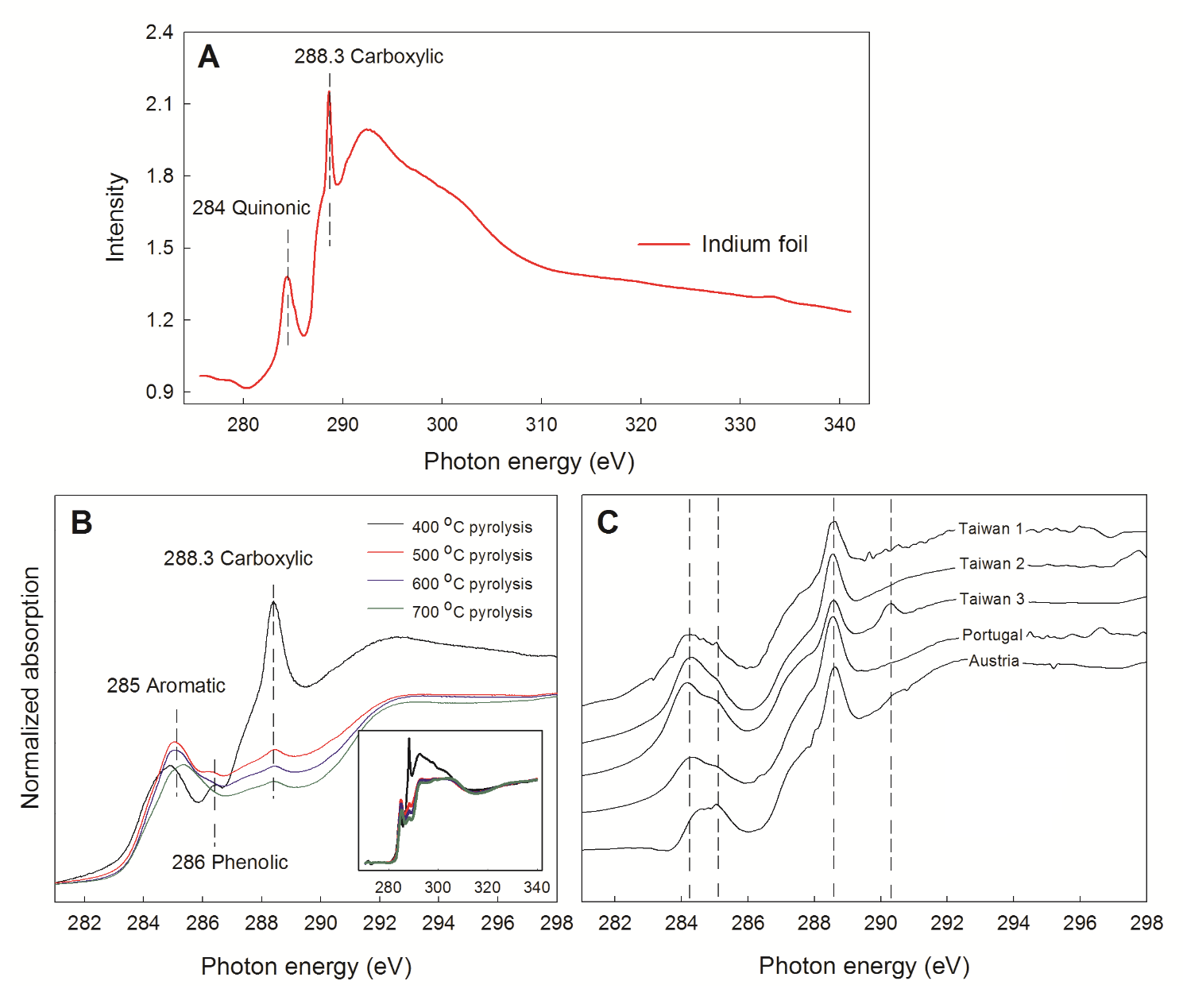


**Fig. SM1.** C NEXAFS spectra for indium foil (99.9975% purity) and samples attached to indium foil that was analyzed at NSRRC Beamline 24A1. (A) Indium foil that was used as sample holder. (B) Biochars that underwent pyrolysis at 400, 500, 600 and 700 °C. (C) Clay fractions extracted from other non-allophanic soils from Taiwan, Portugal, and Austria. Spectral features identified by the vertical lines in inset C correspond to carbon in (a) quinonic (284.3 eV), (b) aromatic (285 eV), (c) carboxylic/carbonyl (288.6 eV), and (e) carbonyl/carbonate (290.5 eV) functional groups. Note that there were no cartographic shifts of spectra in graph B to observe the difference of intensity of X-ray absorbance between samples, but spectra were slightly shifted in graph C to show those spectra clearly.

**References**

1. Hewitt, A. E., Balks, M. R. & Lowe, D. J. Allophanic Soils in *The Soils of Aotearoa New Zealand* 21-39 (Springer International Publishing, 2021).
2. Pullar, W. A. & Birrell, K. S. Parent materials of Tirau silt loam. *New Zealand Journal of Geology and Geophysics,* **16**: 677−686 (1973).
3. Lowe, D. J. Controls on the rates of weathering and clay mineral genesis in airfall tephras: a review and New Zealand case study. In: S.M. Colman and D.P. Dethier (Editors), *Rates of Chemical Weathering of Rocks and Minerals*. Academic Press, Orlando, pp. 265−330 (1986).
4. Lowe, D. J. Stratigraphy, age, composition, and correlation of late Quaternary tephras interbedded with organic sediments in Waikato lakes, North Island, New Zealand. *New Zealand Journal of Geology and Geophysics*, **31**: 125−165 (1988).
5. Lowe, D. J., Neall, V. E., Hedley, M., Clothier, B., Mackay, A. Guidebook for Pre-conference North Island, New Zealand ‘Volcanoes to Ocean’ field tour, 19^th^ World Congress of Soil Science, Brisbane. *Massey University Soil and Earth Sciences Occasional Publication*, **3**, pp. 1−239 (2010).
6. Lowe, D. J., Lanigan, K. M., Palmer, D. J. Where geology meets pedology: late Quaternary tephras, loess, and paleosols in the Mamaku Plateau and Lake Rerewhakaaitu areas. *Geoscience Society of New Zealand Miscellaneous Publication,* **134B**, pp. 2.1−2.45 (2012).
7. Lowe, D. J., Blaauw, M., Hogg, A. G. & Newnham, R. M. Ages of 24 widespread tephras erupted since 30,000 years ago in New Zealand, with re-evaluation of the timing and palaeoclimatic implications of the Lateglacial cool episode recorded at Kaipo bog. *Quaternary Science Reviews* **74**, 170−194 (2013).
8. Peti, L., Hopkins, J. L., Augustinus, P. Revised tephrochronology for key tephras in the 130-ka Ōrākei Basin maar core, Auckland Volcanic Field, New Zealand: implications for the timing of climatic changes, New Zealand. *New Zealand Journal of Geology and Geophysics,* **64**: 235−249 (2021).
9. Soil Survey Staff. *Keys to Soil Taxonomy, twelth edn*, 362 pp. (USDA Natural Resources Conservation Service, 2014).
10. Leathwick, J., Wilson, G., Rutledge, D., Wardle, P., Morgan, F., Johnston, K., McLeod, M., Kirkpatrick, R. *Land Environments of New Zealand*. Ministry for the Environment, Wellington, and Manaaki Whenua Landcare Research, Hamilton. 184 pp (2003).
11. Valentine, K. W. G. and Dalrymple, J. B. Quaternary buried paleosols: a critical review. *Quaternary Research*, **6**: 209−222 (1976).
12. Bronger, A., Catt, J. A. Paleosols: problems of definition, recognition and interpretation. *Catena Supplement*, **16**: 1−7 (1989).
13. Nairn, I. A., Kobayashi, T., Nakagawa, M. The ~10 ka multiple vent pyroclastic eruption sequence at Tongariro Volcanic Centre, Taupo Volcanic Zone, New Zealand: Part 1. Eruptive processes during regional extension. *Journal of Volcanology and Geothermal Research,* **86**: 19−44 (1998).
14. Lowe, D. J. & Pittari, A. The Taupō eruption sequence of AD 232 ± 10 in Aotearoa New Zealand: a retrospection. *Journal of Geography* *(Chigaku Zasshi)* **130**, 117−141 (2021).
15. Topping, W. W. Tephrostratigraphy and chronology of late Quaternary eruptives from the Tongariro Volcanic Centre, New Zealand. *New Zealand Journal of Geology and Geophysics,* **16**: 397−423 (1973).
16. Donoghue, S. L., Neall, V. E., Palmer, A. S. Stratigraphy and chronology of late Quaternary andesitic tephra deposits, Tongariro Volcanic Centre, New Zealand. *Journal of the Royal Society of New Zealand*, **25**: 115−206 (1995).
17. Moebis, A., Cronin, S. J., Neal, V. E., Smith, I. E. M. Unravelling a complex volcanic history from fine-grained, intricate Holocene ash sequences at the Tongariro Volcanic Centre, New Zealand. *Quaternary International*, **246**: 352−363 (2011).
18. Hogg, A. G., Lowe, D. J., Palmer, J. G., Boswijk, G., Bronk Ramsey, C. J. Revised calendar date for the Taupo eruption derived by ^14^C wiggle-matching using a New Zealand kauri ^14^C calibration data set. *The Holocene*, **22**: 439−449 (2012).
19. Hogg, A. G., Wilson, C. J. N., Lowe, D. J., Turney, C. S. M., White, P., Lorrey, A .M., Manning, S.W., Palmer, J. G., Bury, S., Brown, J., Southon, J., Petchey, F. Wiggle-match radiocarbon dating of the Taupo eruption. *Nature Communications,* **10**: 4669 (2019).
20. [Voloschina, M](https://www.mendeley.com/authors/57203974074)., Lube, G., Procter, J., Meobis, A., Timm, C. [Lithosedimentological and tephrostratigraphical characterisation of small-volume, low-intensity eruptions: the 1800 years Tufa Trig Formation, Mt. Ruapehu (New Zealand)](https://www.mendeley.com/catalogue/c3dac6b4-6e3e-35ab-866a-abea6f3bbfa1/). *Journal of Volcanology and Geothermal Research*, **402**: 106987 (2020).
21. Hewitt, A. E., Balks, M. R. & Lowe, D. J. Pumice Soils in *The Soils of Aotearoa New Zealand* 179−198 (Springer International Publishing, 2021).
22. Soil Survey Staff. Buried soils and their effect on taxonomic classification, *USDA Natural Resources Conservation Service, Soil Survey Technical Note* No. **10**. <https://directives.sc.egov.usda.gov/OpenNonWebContent.aspx?content=29749.wba (2011).
23. McDaniel, P. A., Lowe, D. J., Arnalds, O. & Ping, C.-L. Andisols in *Handbook of Soil Sciences, second edn, Vol. 1: Properties and Processes* (eds Huang, P. M., Li, Y. & Sumner, M. E.) 33.29−33.48 (CRC Press (Taylor & Francis), 2012).
24. Cole, J. W. Description and correlation of Holocene volcanic formations in the Tarawera- Rerewhakaaitu region. *Transactions of the Royal Society of New Zealand (Earth Sciences),* **8**: 93−108 (1970).
25. Hartemink, A. E., Zhang, Y., Bockheim, J. G., Curi, N., Silva, S. H. G., Grauer-Gray, J., Lowe, D. J., Krasilnikov, P. Soil horizon variation: a review. *Advances in Agronomy,* **160**: 125−185 (2020).
